# Comorbidities and HIV‐related factors associated with mental health symptoms and unhealthy substance use among older adults living with HIV in low‐ and middle‐income countries: a cross‐sectional study

**DOI:** 10.1002/jia2.26434

**Published:** 2025-03-05

**Authors:** Jeremy L. Ross, Dhanushi Rupasinghe, Thida Chanyachukul, Brenda Crabtree Ramírez, Gad Murenzi, Edith Kwobah, Fiona Mureithi, Albert Minga, Ivan Marbaniang, Hugo Perazzo, Angela Parcesepe, Suzanne Goodrich, Cleophas Chimbetete, Ephrem Mensah, Fernanda Maruri, Dung Thi Hoai Nguyen, Alvaro López‐Iñiguez, Kathryn Lancaster, Helen Byakwaga, Mpho Tlali, Marie K. Plaisy, Smita Nimkar, Rodrigo Moreira, Kathryn Anastos, Aggrey Semeere, Gilles Wandeler, Antoine Jaquet, Annette Sohn, the Sentinel Research Network of the International epidemiology Databases to Evaluate AIDS

**Affiliations:** ^1^ TREAT Asia/amfAR – The Foundation for AIDS Research Bangkok Thailand; ^2^ The Kirby Institute UNSW Sydney Sydney New South Wales Australia; ^3^ Departamento de Infectología Instituto Nacional de Ciencias Médicas y Nutrición México City México; ^4^ Research for Development (RD Rwanda) Kigali Rwanda; ^5^ AMPATH MOI University Eldoret Kenya; ^6^ Infectious Disease Research in Zambia (CIDRZ) Lusaka Zambia; ^7^ The HIV care clinic of the National Blood Transfusion Centre Blood Bank Medical Centre Abidjan Côte d'Ivoire; ^8^ BJ Government Medical College‐JHU Clinical Research Site Pune India; ^9^ Instituto Nacional de Infectologia Evandro Chagas (INI) Fundação Oswaldo Cruz (FIOCRUZ) Rio de Janeiro Brazil; ^10^ Gillings School of Global Public Health University of North Carolina at Chapel Hill Chapel Hill North Carolina USA; ^11^ Division of Infectious Diseases Indiana University School of Medicine Indianapolis Indiana USA; ^12^ Newlands Clinic Harare Zimbabwe; ^13^ NGO Espoir‐Vie Togo Lomé Togo; ^14^ Division of Infectious Diseases Department of Medicine, Vanderbilt University Medical Center Nashville Tennessee USA; ^15^ National Hospital for Tropical Diseases Hanoi Vietnam; ^16^ Division of Public Health Sciences Wake Forest University School of Medicine Winston‐Salem North Carolina USA; ^17^ Mbarara ISS Clinic Mbarara Uganda; ^18^ Centre for Infectious Disease Epidemiology & Research, School of Public Health University of Cape Town Cape Town South Africa; ^19^ National Institute for Health and Medical Research (INSERM) UMR 1219, Research Institute for Sustainable Development (IRD) EMR 271 University of Bordeaux, Bordeaux Population Health Centre Bordeaux France; ^20^ Montefiore Medical Center Albert Einstein College of Medicine New York New York USA; ^21^ Infectious Diseases Institute Kampala Uganda; ^22^ Department of Infectious Diseases Bern University Hospital, University of Bern Bern Switzerland

**Keywords:** ageing, coinfections, comorbidities, HIV, mental health, substance use

## Abstract

**Introduction:**

People with HIV (PWH) are vulnerable to mental health and substance use disorders (MSDs), but the extent to which these are associated with other non‐communicable diseases in ageing PWH populations remains poorly documented. We assessed comorbidities associated with symptoms of MSD among PWH ≥40 years in the Sentinel Research Network (SRN) of the International epidemiology Database to Evaluate AIDS (IeDEA).

**Methods:**

Baseline data collected between June 2020 and September 2022, from 10 HIV clinics in Asia, Latin America and Africa contributing to the SRN, were analysed. Symptoms of MSDs and comorbidities were assessed using standardized questionnaires, anthropometric and laboratory tests, including weight, height, blood pressure, glucose, lipids, chronic viral hepatitis and liver transient elastography. HIV viral load, CD4 count and additional routine clinical data were accessed from participant interview or medical records. HIV and non‐HIV clinical associations of mental illness symptoms and unhealthy substance use were analysed using logistic regression. Mental illness symptoms were defined as moderate‐to‐severe depressive symptoms (PHQ‐9 score >9), moderate‐to‐severe anxiety symptoms (GAD‐7 >9) or probable post‐traumatic stress disorder (PCL‐5 >32). Unhealthy substance use was defined as ASSIST score >3, or AUDIT ≥7 for women (≥8 for men).

**Results:**

Of 2614 participants assessed at baseline study visits, 57% were female, median age was 50 years, median CD4 was 548 cells/mm^3^ and 86% had HIV viral load <1000 copies/ml. Overall, 19% had mental illness symptoms, 15% unhealthy substance use, 49% BMI >25 kg/m^2^, 38% hypertension, 15% type 2 diabetes, 35% dyslipidaemia, 34% liver disease and 23% history of tuberculosis. BMI >25 and dyslipidaemia were found in 54% and 40% of those with mental illness symptoms compared to 49% and 34% of those without. Mental illness symptoms were not significantly associated with the clinical factors assessed. Unhealthy substance use was more likely among those with dyslipidaemia (OR 1.55, CI 1.16−2.09, *p* = 0.003), and less likely in those with BMI >25 (OR 0.48, CI 0.30−0.77, *p* = 0.009).

**Conclusions:**

Improved integration of MSD and comorbidity services in HIV clinical settings, and further research on the association between MSD and comorbidities, and care integration among older PWH in low‐middle‐income countries, are required.

## 1 INTRODUCTION

Expanded coverage of increasingly effective combination antiretroviral therapy (ART) has reduced mortality among people with HIV (PWH), improved life expectancy, and shifted focus towards the management of HIV as a chronic disease [1, 2, 3]. In the United States and Europe, improved survival of younger PWH or increasing rates of HIV acquisition among older people have contributed to an increased proportion of PWH aged ≥ 50 years old [4]. UNAIDS estimates the number of people aged 50 years or older with HIV globally, increased from 5.4 million in 2015 to 8.1 million in 2020 [5], and nearly 75% of all PWH will be aged 50 years or older by 2030 [6].

Older PWH experience a number of health challenges including ageing, functional decline, non‐communicable diseases (NCDs), multimorbidity and polypharmacy [7, 8, 9, 10]. Older PWH have a higher risk and prevalence of NCDs, including cardiovascular, metabolic and hepatic disease, and are also at increased risk of coinfections such as tuberculosis (TB) and viral hepatitis, compared to younger PWH or those without HIV [11, 12, 13, 14, 15, 16].

The burden of mental health and substance use disorders (MSDs) among adult PWH is high, with rates often higher among those living with HIV than those without [17, 18, 19, 20]. Rates of certain mental health disorders are higher among older PWH compared to younger PWH, and substance use among PWH does not decline with increasing age [21, 22]. Depression, anxiety and post‐traumatic stress disorder (PTSD) are prevalent among adult PWH, commonly co‐occurring with substance use [23, 24, 25, 26]. In low‐ and middle‐income countries (LMICs), high rates of mental health disorders and substance use have been documented [19]. MSDs among adult PWH are associated with several negative HIV‐related and other health impacts, including mortality, suboptimal ART adherence, poorer engagement in HIV care and virologic failure [27, 28, 29, 30, 31, 32, 33, 34].

Improved understanding of clinical factors associated with MSDs and their interactions might guide interventions among older PWH at risk of MSDs, NCDs and co‐infections. We, therefore, analysed HIV and non‐HIV‐related clinical factors associated with recent mental illness symptoms or unhealthy substance use among participants enrolled in a multiregional cohort of older PWH. HIV‐related clinical factors included ART regimen, CD4 count and viral load. Non‐HIV clinical factors included comorbid NCDs such as cardiometabolic and liver disease, and co‐infections such as chronic viral hepatitis and TB. We hypothesized that mental illness symptoms and unhealthy substance use would be associated with non‐nucleoside reverse transcriptase inhibitor (NNRTI)‐based ART (still commonly used in LMICs), lower CD4 count, detectable viral load, comorbid NCDs and coinfections in our cohort.

## 2 METHODS

This study utilized data from the Sentinel Research Network (SRN), a prospective cohort within the International epidemiology Database to Evaluate AIDS (IeDEA) consortium [35]. The SRN comprises 2925 PWH aged ≥ 40 years, on ART for at least 6 months at their baseline study visit. Twelve HIV clinics in 12 countries and six global IeDEA regions currently participate in SRN: Asia‐Pacific (*n* = 2: India, Vietnam), Caribbean, Central and South America (*n* = 2: Brazil, Mexico), Central Africa (*n* = 1: Rwanda), East Africa (*n* = 3: Kenya, Tanzania, Uganda), Southern Africa (*n* = 2: Zambia, Zimbabwe) and West Africa (*n* = 2: Cote d'Ivoire, Togo). SRN participants undergo baseline (enrolment), 6‐, 12‐, 24‐ and 36‐month study visits, during which standardized questionnaires and non‐to‐minimally invasive screening tests assess various chronic conditions.

The Alcohol Use Disorders Identification Test (AUDIT) is used to assess alcohol consumption; the Alcohol, Smoking and Substance Involvement Screening Test (ASSIST) for substance use (tobacco, alcohol, cannabis, cocaine, amphetamine‐type stimulants, inhalants, sedatives, hallucinogens, opioids) [36]; the Patient Health Questionnaire (PHQ‐9) for depression; the Generalized Anxiety Disorder 7‐item (GAD‐7) scale for anxiety; and the post‐traumatic stress disorder (PTSD) Checklist for DSM‐5 (PCL‐5) to assess PTSD. Physical activity is assessed using the WHO Global Physical Activity Questionnaire (GPAQ). Weight, height, waist and hip circumference, and blood pressure measurements are taken. Laboratory or point‐of‐care tests are performed for glycosylated haemoglobin (HbA1c), fasting plasma glucose (FPG), fasting lipids, complete blood count, platelets, transaminases (AST/ALT), creatinine, and hepatitis B and C. Additionally, participants are screened for liver fibrosis and steatosis using transient elastography (Fibroscan®). HIV‐related participant data, including ART, HIV viral load and CD4, are accessed from regional IeDEA databases. Medical histories and additional laboratory data are obtained from participant interviews and site medical records review.

We conducted a cross‐sectional analysis of SRN baseline data from study participants completing PHQ‐9, GAD‐7, PCL‐5, AUDIT, ASSIST and GPAQ screenings between June 2020 and October 2022 at 10 SRN sites in 10 countries (Brazil, Cote d'Ivoire, India, Kenya, Mexico, Rwanda, Togo, Uganda, Zambia and Zimbabwe). Patients with missing responses to these screenings were included in the analysis with missing responses imputed using the “hot deck” imputation method [37]. Factors associated with a combined mental illness symptom outcome, and a combined unhealthy substance use outcome were analysed separately using logistic regression. The combined outcome of mental illness symptoms was defined as having moderate‐to‐severe depressive symptoms (PHQ‐9 score of >9), moderate‐to‐severe anxiety symptoms (GAD‐7 score of >9) or provisional diagnosis of PTSD (PCL‐5 score >32). Unhealthy substance use was defined as having an ASSIST Specific Substance Involvement score >3 (excluding for alcohol and tobacco) [36, 38, 39] or unhealthy alcohol use (AUDIT score of ≥7 for women, ≥8 for men).

Type 2 diabetes, dyslipidaemia, hypertension or liver disease were defined as currently or ever having these conditions on study test results (clinical measurement or laboratory), self‐reported conditions or medical record review. Past history of TB was through self‐report or evidence of TB treatment on medical record review. The following study test definitions were used: type 2 diabetes—FPG ≥7 mmol/l or HbA1c ≥ 6.5%; dyslipidaemia—LDL cholesterol >4.13 mmol/l, total cholesterol ≥6.2 mmol/l or triglycerides >2.25 mmol/l; hypertension—systolic blood pressure ≥140 mmHg or diastolic blood pressure ≥90 mmHg from an average of three measures on the same day; liver disease—ALT >5 times its upper limit of normal (ULN) value of 40 IU/l, AST >5 times ULN of 40 IU/l, positive HBsAg test, positive anti‐hepatitis C virus (HCV) test, liver stiffness measurement (LSM) ≥7.1KPA or Controlled Attenuation Parameter (CAP) ≥248 dB/m. Body mass index (BMI) was categorized as underweight (<18 kg/m^2^), healthy weight (18–24.9 kg/m^2^), overweight (25–29.9 kg/m^2^) or obesity (≥30 kg/m^2^). Inadequate physical activity was defined as not achieving WHO physical activity recommendations of 150 minutes of moderate‐intensity physical activity, 75 minutes of vigorous‐intensity physical activity or an equivalent combination of moderate‐ and vigorous‐intensity physical activity achieving at least 600 metabolic equivalent tasks‐minutes in a week. Detectable viral load was defined as >1000 copies/ml [40].

Covariates in the univariate analysis with *p* < 0.10 were fitted into the multivariate model. Backward‐stepwise selection process was used, and covariates with *p*<0.05 were considered statistically significant and retained in the multivariate model. SAS Enterprise guide (SAS Institute Inc., Cary, NC, USA) and Stata software version 16.1 (StataCorp, College Station, TX, USA) were used to perform all data management and statistical analyses.

The SRN study has been performed in accordance with the Declaration of Helsinki. All participants were informed about the benefits and potential harms related to their participation. Study participants were consented using standard informed consent and study information forms. All participating study sites, and coordinating and data management centres obtained institutional review board (IRB) approvals for participation: INI‐Fiocruz, Brazil: 28609820.9.0000.5262; CMSDS, Côte d'Ivoire: 195‐21; BJ Medical College, India: 00241024; AMPATH Eldoret, Kenya: 0003638; INCMNSZ, Mexico: 3708; Kicukiro Health Center, Rwanda: 885/RNEC/2022; EVT Clinic, Togo: 01/2022/CBRS; Mbarara University of Science and Technology, Uganda: MUST‐2022‐379, and Uganda National Council for Science and Technology: HS2217ES; CIDRZ, Zambia; 00001131 of IORG0000774; Medical Research Council of Zimbabwe: MRCZ/A/2475.

## 3 RESULTS

### 3.1 Participant characteristics

A total of 2614 participants who had completed the PHQ‐9, GAD‐7, PCL‐5, AUDIT, ASSIST and GPAQ screenings at baseline study visits conducted between June 2020 and September 2022 were included (Table 1). These included two participants with at least one missing response in at least one of these screenings.

**Table 1:** Participant characteristics at enrolment into SRN (baseline visit)

	Total participants	Participants with mental illness symptoms	Participants with unhealthy substance use
**Total**	2614 (100)	508 (19)	394 (15)
**Sex**			
Male	1126 (43)	184 (36)	269 (68)
Female	1488 (57)	324 (64)	125 (32)
**Age (years)**			
Median (IQR)	50 (45–56)	50 (45–54)	49 (44–53)
40–50	1243 (48)	222 (44)	153 (39)
≥50	1371 (52)	286 (56)	241 (61)
**Marital status**			
Single	469 (18)	134 (26)	106 (27)
Married	1149 (44)	162 (32)	158 (40)
Widowed	547 (21	112 (22)	40 (10)
Separated	102 (4)	10 (2)	22 (6)
Divorced	178 (7)	57 (11)	34 (9)
Living with partner	165 (6)	33 (7)	34 (9)
Missing	4 (0)	0 (0)	0 (0)
**Education**			
None	240 (9)	63 (12)	34 (9)
Primary education	870 (33)	165 (32)	108 (27)
Lower secondary or end of basic education	707 (27)	125 (25)	98 (25)
Upper secondary or post‐secondary non‐tertiary	430 (16)	83 (16)	67 (17)
University or post‐graduate	345 (13)	68 (13)	86 (22)
Other/Don't know/Missing	22 (1)	4 (0)	1 (0)
**Monthly income**			
< 40 USD	469 (18)	107 (21)	59 (15)
≥40 and <80 USD	678 (26)	123 (24)	78 (20)
≥80 and < 200 USD	688 (26)	119 (23)	93 (24)
≥200 and <400 USD	369 (14)	78 (15)	66 (17)
≥400 USD	332 (13)	61 (12)	87 (22)
Don't know/Missing	78 (3)	19 (4)	11 (3)
**CD4 count (cells/mm^3^) at SRN enrolment**			
Median (IQR)	548 (383–736)	552 (380–747)	537 (370–785)
<200	125 (5)	34 (7)	25 (6)
200–349	318 (12)	59 (12)	53 (14)
350–499	477 (18)	90 (18)	78 (20)
≥500	1263 (48)	257 (51)	200 (51)
Not reported	431 (17)	68 (13)	38 (10)
**Viral load (copies/ml) at SRN enrolment**			
Median (IQR)	39 (20–60)	40 (20–93)	40 (20–60)
<1000	2235 (86)	441 (87)	355 (90)
≥1000	123 (5)	27 (5)	19 (5)
Not reported	256 (10)	40 (8)	20 (5)
**Current ART regimen**			
INSTI‐based	1123 (43)	198 (39)	162 (41)
PI‐based	220 (9)	58 (11)	29 (7)
NNRTI‐based	864 (33)	147 (29)	114 (29)
Other	407 (15)	105 (21)	89 (23)
**BMI** (**kg/m^2^)**			
Median (IQR)	25 (22–29)	25 (21–30)	24 (21–29)
<18 (Underweight)	174 (7)	37 (7)	29 (7)
18–24.9 (Healthy weight)	1143 (44)	197 (39)	187 (47)
25–29.9 (Overweight)	795 (30)	163 (32)	109 (28)
≥30 (Obese)	502 (19)	111 (22)	69 (18)
**Hypertension**			
No	1618 (62)	320 (63)	250 (63)
Yes	996 (38)	188 (37)	144 (37)
**Diabetes**			
No	2218 (85)	426 (84)	339 (86)
Yes	396 (15)	82 (16)	55 (14)
**Dyslipidaemia**			
No	1701 (65)	307 (60)	217 (55)
Yes	913 (35)	201 (40)	177 (45)
**Liver disease**			
No	1721 (66)	323 (64)	260 (66)
Yes	893 (34)	185 (36)	134 (34)
**Tuberculosis**			
No	2006 (77)	391 (77)	292 (74)
Yes	608 (23)	117 (23)	102 (26)
**Adequate physical activity**			
No	2523 (97)	493 (97)	378 (96)
Yes	91 (4)	15 (3)	16 (4)
**Region**			
Asia‐Pacific	200 (8)	13 (3)	6 (2)
Caribbean, Central and South America	420 (16)	135 (27)	116 (29)
Central Africa	596 (23)	100 (20)	54 (14)
East Africa	300 (11)	34 (7)	25 (6)
Southern Africa	498 (19)	92 (18)	77 (20)
West Africa	600 (23)	134 (26)	116 (29)

Abbreviations: ART, antiretroviral therapy; BMI, body mass index; IQR, interquartile range; MSM, men who have sex with men.

The majority were female at birth (*n* = 1488, 57%) with a median age of 50 years (interquartile range [IQR] 45–56). Most were married (*n* = 1149, 44%), with primary (*n* = 870, 33%) or lower secondary education (*n* = 707, 27%), and a monthly income < 200 USD (*n* = 1835, 70%). The median CD4 cell count was 548 cells/mm^3^ (IQR 383–736), and most (*n* = 2235, 86%) had an HIV viral load < 1000 copies/ml. At the time of SRN baseline visit, 43% (*n* = 1123) were on an integrase strand inhibitor (INSTI)‐containing ART regimen, 33% (*n* = 864) on an NNRTI regimen and 9% (*n* = 220) on a protease inhibitor (PI) regimen. Among those on “other” ART, most were on mono/dual regimens (*n* = 184) or NRTIs (*n* = 94).

The median BMI at enrolment was 25 kg/m^2^ (IQR 22–29) and 49% (*n* = 1297) had a BMI ≥25 kg/m^2^. At the baseline study visit, 38% (*n* = 996) had hypertension, 15% (*n* = 396) had type 2 diabetes, 35% (*n* = 913) had dyslipidaemia, 34% (*n* = 893) had liver disease, 23% (*n* = 608) had a history of TB and 97% (*n* = 2523) had inadequate physical activity. Of the 2614 study participants, 19% (*n* = 508) had recent mental illness symptoms and 15% (*n* = 394) had unhealthy substance use (Table 1). Among those with baseline mental illness symptoms or unhealthy substance use, most were from the Caribbean, Central and South America, West Africa, Asia‐Pacific, Central Africa and Southern Africa regions. The most common substances used among those with unhealthy substance use were alcohol (*n* = 297), sedatives (*n* = 60), cannabis (*n* = 48), cocaine (*n* = 21) and amphetamines (*n* = 13).

### 3.2 Clinical factors among those with and without mental illness symptoms and unhealthy substance use

**Figure 1:**
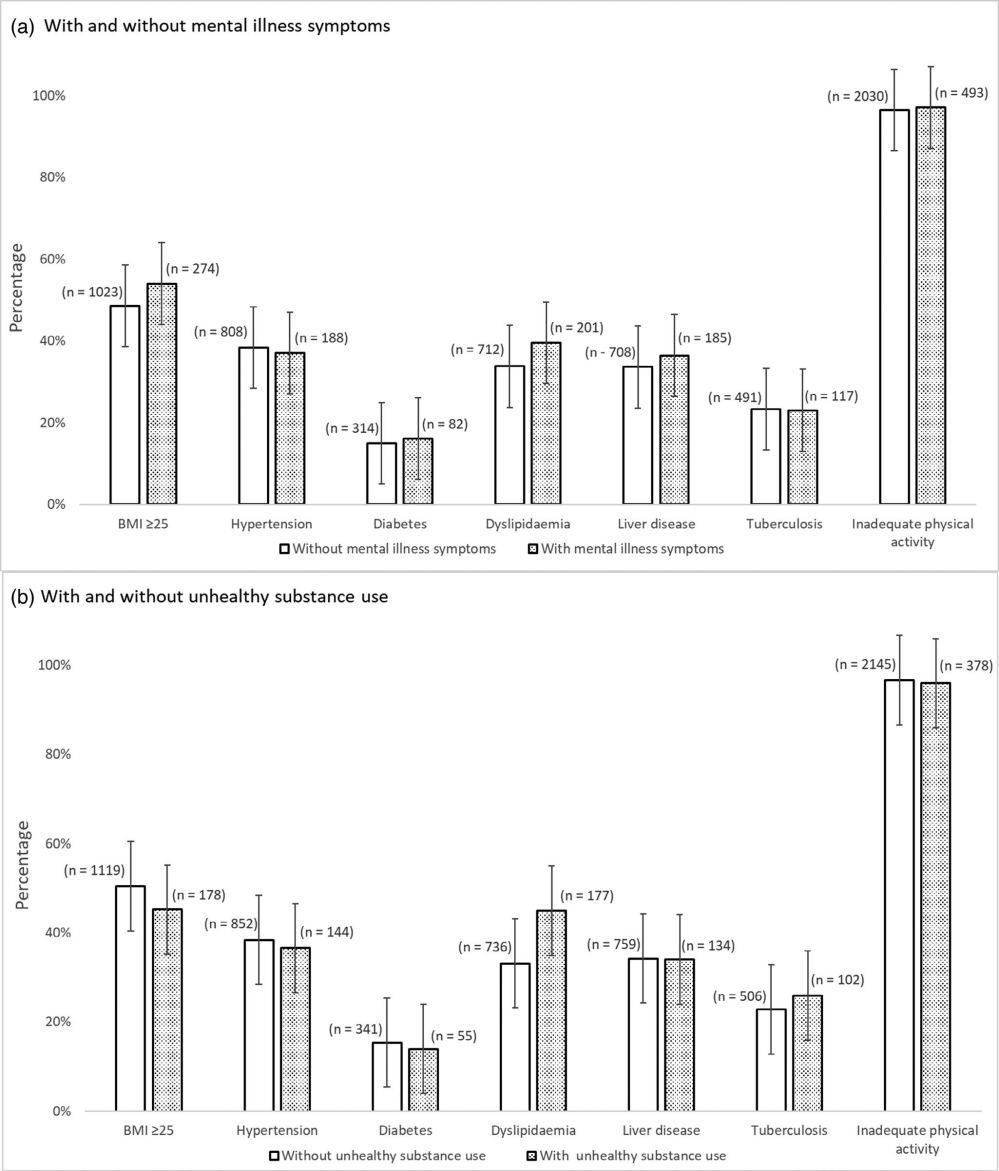
Clinical factors among participants. Bar chart of numbers and proportions of clinical factors among participants with and without symptoms of mental illness and unhealthy substance use.

A higher proportion of individuals with mental illness symptoms had BMI ≥25 kg/m^2^ (54% vs. 49%, *p* = 0.015) and dyslipidaemia (40% vs. 34%, *p* = 0.007), than those without mental illness symptoms (Figure 1a). The prevalence of hypertension, type 2 diabetes, liver disease, history of TB and inadequate physical activity were similar in those with mental illness symptoms, compared to those without.

Dyslipidaemia was more prevalent in those with unhealthy substance use compared to those without unhealthy substance use (45% vs. 33%, *p*<0.001). BMI>25 kg/m^2^ was less prevalent among those with unhealthy substances compared to those without (46% vs. 51%, *p* = 0.027) (Figure 1b). The prevalence of hypertension, type 2 diabetes, liver disease, history of TB and inadequate physical activity were similar in those with unhealthy substance use compared to those without.

### 3.3 Factors associated with recent mental illness symptoms

No association was found between mental illness symptoms, and viral load, CD4 count, overweight or obese BMI, hypertension, type 2 diabetes, dyslipidaemia, liver disease, history of TB or adequate physical activity (Table 2).

**Table 2:** Factors associated with recent mental illness symptoms

	No. of patients	No. of events	Univariate			Multivariable		
			OR	95% CI	*p*‐value	OR	95% CI	*p*‐value
**Total**	2614 (100)	508 (19)						
**Sex**								
Male	1126 (43)	184 (36)	1			1		
Female	1488 (57)	324 (64)	1.76	(1.41, 2.19)	**<0.001**	1.75	(1.40, 2.18)	<0.001
**Age (years)**								
40–50	1243 (48)	222 (44)	1			1		
≥50	1371 (52)	286 (56)	1.29	(1.05, 1.57)	**0.013**	1.23	(1.01, 1.51)	0.042
**CD4 count (cells/mm^3^)**					0.232			
<200	125 (5)	91 (4)	1					
200–349	318 (12)	259 (12)	0.65	(0.39, 1.06)	0.084			
350–499	477 (18)	387 (18)	0.63	(0.40, 1.01)	0.054			
≥500	1263 (48)	1006 (48)	0.65	(0.42, 0.99)	0.046			
Not reported	431 (17)	363 (17)						
**Viral load (copies/ml)**								
≤1000	2235 (86)	1794 (85)	1					
>1000	123 (5)	96 (5)	1.23	(0.79, 1.93)	0.363			
Not reported	256 (10)	216 (10)						
**Current ART regimen**					**0.009**			**0.014**
INSTI‐based	1123 (43)	198 (39)	1			1		
PI‐based	220 (9)	58 (11)	1.57	(1.07, 2.28)	0.02	1.42	(0.97, 2.08)	0.07
NNRTI‐based	864 (33)	147 (29)	0.95	(0.68, 1.31)	0.738	0.88	(0.63, 1.22)	0.434
Other	407 (15)	105 (21)	1.35	(1.01, 1.81)	0.045	1.34	(1.00, 1.81)	0.05
**BMI (kg/m^2^)**					0.131			
<18 (Underweight)	174 (7)	37 (7)	1					
18–24.9 (Healthy weight)	1143 (44)	197 (39)	0.64	(0.43, 0.96)	0.031			
25–29.9 (Overweight)	795 (30)	163 (32)	0.7	(0.46, 1.06)	0.096			
≥30 (Obesity)	502 (19)	111 (22)	0.78	(0.50, 1.20)	0.252			
**Hypertension**								
No	1618 (62)	320 (63)	1					
Yes	996 (38)	188 (37)	0.88	(0.72, 1.09)	0.242			
**Diabetes**								
No	2218 (85)	426 (84)	1					
Yes	396 (15)	82 (16)	1.15	(0.87, 1.51)	0.324			
**Dyslipidaemia**								
No	1701 (65)	307 (60)	1					
Yes	913 (35)	201 (40)	1.00	(0.77, 1.29)	0.987			
**Liver disease**								
No	1721 (66)	323 (64)	1					
Yes	893 (34)	185 (36)	0.96	(0.77, 1.19)	0.712			
**Tuberculosis**								
No	2006 (77)	391 (77)	1					
Yes	608 (23)	117 (23)	1.13	(0.89, 1.44)	0.32			
**Adequate physical activity**								
No	2523 (97)	2030 (96)	1					
Yes	91 (4)	76 (4)	0.75	(0.42, 1.34)	0.333			
**Region**					**<0.001**			**<0.001**
Asia‐Pacific	200 (8)	13 (23)	1			1		
Caribbean, Central and South America	420 (16)	135 (27)	6.81	(3.75, 12.39)	<0.001	7.41	(4.04, 13.59)	<0.001
Central Africa	596 (23)	100 (20)	2.9	(1.59, 5.29)	0.001	3.05	(1.65, 5.64)	<0.001
East Africa	300 (11)	34 (7)	1.84	(0.94, 3.58)	0.073	1.6	(0.80, 3.18)	0.183
Southern Africa	498 (19)	92 (18)	3.26	(1.78, 5.98)	<0.001	2.94	(1.57, 5.48)	0.001
West Africa	600 (23)	134 (26)	4.14	(2.28, 7.49)	<0.001	3.65	(1.97, 6.76)	<0.001

*Note*: Covariates with univariable association have been included in the multivariable model.

Bold numbers are supposed to denote statisitically significant *p*‐values.

Abbreviations: ART, antiretroviral therapy; BMI, body mass index; MSM, men who have sex with men; No., number; OR, odds ratio.

Mental illness symptoms were more likely in those on “other” ART (OR 1.34, confidence interval [CI] 1.00–1.81, *p* = 0.05) compared to those on INSTI‐based ART. Mental illness symptoms were more likely in females (OR 1.75, CI 1.40–2.18, *p*<0.001) compared to males, and those ≥ 50 years old (OR 1.23, CI 1.01–1.51, *p* = 0.042) compared to those <50 years. Mental illness symptoms were also more likely in other regions (Caribbean, Central and South America: OR 7.41, CI 4.04–13.59, *p*<0.001; West Africa: OR 3.65, CI 1.97–6.76, *p*<0.001; Central Africa: OR 3.05, CI 1.65–5.64, *p* = 0.001; Southern Africa: OR 2.94, CI 1.57–5.48, *p*<0.001) compared to the Asia‐Pacific.

### 3.4 Factors associated with unhealthy substance use

Unhealthy substance use was more likely among those with dyslipidaemia (OR 1.55, CI 1.16–2.09, *p* = 0.003) compared to those without dyslipidaemia, aged 50 years and older (OR 1.93, CI 1.53–2.44, *p*<0.001) compared to 40–50 years, and in other regions (West Africa: OR 15.66, CI 6.55–37.39, *p*<0.001; Caribbean, Central and South America: OR 12.24, CI 5.02–28.84, *p*<0.001; Southern Africa: OR 11.27, CI 4.65–27.33, *p*<0.001; Central Africa: OR 5.02, CI 2.14–12.66, *p*<0.001; East Africa: OR 4.96, CI 1.94–12.83, *p* = 0.001) compared to the Asia‐Pacific (Table 3).

**Table 3:** Factors associated with unhealthy substance use

	No. of patients	No. of events	Univariate			Multivariable		
			OR	95% CI	*p*‐value	OR	95% CI	*p*‐value
**Total**	2614 (100)	394 (15)						
**Sex**								
Male	1126 (43)	269 (68)	1			1		
Female	1488 (57)	125 (32)	0.29	(0.23, 0.37)	<0.001	0.28	(0.21, 0.36)	**<0.001**
**Age (years)**								
40–50	1243 (48)	153 (39)	1			1		
≥50	1371 (52)	241 (61)	1.63	(1.30, 2.04)	<0.001	1.93	(1.53, 2.44)	**<0.001**
**CD4 count (cells/mm^3^)**					0.439			
<200	125 (5)	25 (6)	1					
200–349	318 (12)	53 (14)	0.88	(0.51, 1.51)	0.644			
350–499	477 (18)	78 (20)	0.82	(0.49, 1.38)	0.455			
≥500	1263 (48)	200 (51)	0.73	(0.45, 1.17)	0.19			
Not reported	431 (17)	38 (10)						
**Viral load (copies/ml)**								
≤1000	2235 (86)	355 (90)	1					
>1000	123 (5)	19 (5)	1.12	(0.67, 1.88)	0.671			
Not reported	256 (10)	20 (5)						
**Current ART regimen**					**0.047**			
INSTI‐based	1123 (43)	162 (41)	1					
PI‐based	220 (9)	29 (7)	0.92	(0.58, 1.46)	0.72			
NNRTI‐based	864 (33)	114 (29)	1.28	(0.90, 1.82)	0.163			
Other	407 (15)	89 (23)	1.49	(1.09, 2.03)	0.013			
**BMI (kg/m^2^)**					**<0.001**			**0.038**
<18 (Underweight)	174 (7)	29 (7)	1			1		
18–24.9 (Healthy weight)	1143 (44)	187 (47)	0.73	(0.47, 1.15)	0.175	0.71	(0.45, 1.12)	0.143
25–29.9 (Overweight)	795 (30)	109 (28)	0.48	(0.30, 0.77)	0.002	0.52	(0.32, 0.85)	0.009
≥30 (Obesity)	502 (19)	69 (18)	0.48	(0.29, 0.78)	0.004	0.64	(0.38, 1.08)	0.092
**Hypertension**								
No	1618 (62)	250 (63)	1					
Yes	996 (38)	144 (37)	0.81	(0.64, 1.03)	**0.08**			
**Diabetes**								
No	2218 (85)	339 (86)	1					
Yes	396 (15)	55 (14)	0.90	(0.65, 1.24)	0.517			
**Dyslipidaemia**								
No	1701 (65)	217 (55)	1			1		
Yes	913 (35)	177 (45)	1.33	(1.01, 1.75)	**0.041**	1.55	(1.16, 2.09)	**0.003**
**Liver disease**								
No	1721 (66)	260 (66)	1					
Yes	893 (34)	134 (34)	0.8	(0.62, 1.02)	**0.077**			
**Tuberculosis**								
No	2006 (77)	292 (74)	1					
Yes	608 (23)	102 (26)	1.40	(1.08, 1.82)	**0.011**			
**Adequate physical activity**								
No	2523 (97)	378 (96)	1					
Yes	94 (4)	16 (4)	1.19	(0.67, 2.12)	0.55			
Not reported	1453 (56)	211 (54)						
**Region**					**<0.001**			**<0.001**
Asia‐Pacific	200 (8)	6 (2)	1			1		
Caribbean, Central and South America	420 (16)	116 (29)	12.34	(5.33, 28.58)	<0.001	12.24	(5.20, 28.84)	<0.001
Central Africa	596 (23)	54 (14)	3.22	(1.36, 7.61)	0.008	5.2	(2.14, 12.66)	<0.001
East Africa	300 (11)	25 (6)	2.94	(1.18, 7.30)	0.02	4.96	(1.94, 12.63)	0.001
Southern Africa	498 (19)	77 (20)	5.91	(2.53, 13.81)	<0.001	11.27	(4.65, 27.33)	<0.001
West Africa	600 (23)	116 (29)	7.75	(3.35, 17.90)	<0.001	15.66	(6.55, 37.39)	<0.001

*Note*: Covariates with univariable association have been included in the multivariable model.

Bold numbers are supposed to denote statisitically significant *p*‐values.

Abbreviations: ART, antiretroviral therapy; BMI, body mass index; MSM, men who have sex with men; No., number; OR, odds ratio.

Unhealthy substance use was less likely among those overweight (OR 0.52, CI 0.32–0.85, *p* = 0.009) compared to those underweight, and females (OR 0.28, CI 0.21–0.36, *p*<0.001) compared to males. No association was found between unhealthy substance use and viral load, CD4 count, current ART regimen, hypertension, type 2 diabetes, liver disease, history of TB or adequate physical activity.

## 4 DISCUSSION

In our baseline analysis of 2614 PWH ≥40 years old under care at 10 HIV clinics in Asia, Latin America and Africa, there was a high prevalence of comorbidities. BMI ≥25 kg/m^2^ and dyslipidaemia were more prevalent among those with mental illness symptoms compared to those without. Unhealthy substance use was significantly more likely among those with dyslipidaemia, and less likely among those overweight.

Our finding that mental illness symptoms or unhealthy substance use were not associated with CD4 cell count contrasts with previous research. Depression has been associated with lower CD4 in both high‐income and LMIC PWH cohorts [41, 42, 43]. Substance use, including alcohol and smoking, has been consistently associated with lower CD4 counts, including among older PWH populations in LMICs [44, 45, 46, 47]. Prior research on the association between MSDs and detectable viral load is conflicting, with some finding a positive association [48, 49] but other studies not [31]. Finding no association between mental illness symptoms and NNRTI and INSTI use contrasts with documented neuropsychiatric side effects of efavirenz and dolutegravir [50, 51, 52]. The lack of association between mental illness symptoms, unhealthy substance use and these HIV clinical factors in our cohort might relate to the predominantly urban study sites with relatively well‐resourced programmes for PWH care and follow‐up. Our use of composite mental illness and substance use endpoints covering distinct mental health conditions and substances, and not distinguishing between specific anti‐retroviral drugs might also have contributed.

In our analysis, a history of TB was not significantly associated with mental illness symptoms or unhealthy substance use. Associations between mental health disorders, higher risk of active TB and negative TB treatment outcomes are well‐documented in non‐PWH populations [53, 54, 55], and substance use, particularly alcohol, is associated with TB development and negative TB treatment outcomes among adults with and without HIV [56, 57, 58, 59]. However, associations between MSDs and a history of TB in PWH are less consistent. Some recent analyses in LMIC settings have found comorbid/recent TB or opportunistic infections were associated with MSDs, while others found MSDs were not associated with a prior history [60, 61, 62], suggesting that recency of TB might influence the association. In addition, documenting the past history of TB in our study relied on self‐report or medical record review, with the potential for underreporting.

The higher prevalence of overweight and dyslipidaemia among those with mental illness symptoms in our cohort is consistent with findings associating mood disorders with cardiometabolic NCDs among PWH cohorts in the United States. Among over 4000 older PWH in the United States, mood disorders were associated with an increased risk of first NCD and a 29% increased risk of metabolic syndrome multimorbidity, defined as any three of hypertension, obesity, hyperlipidaemia and diabetes [63]. In a large U.S. cohort, risks for all comorbidities were highest for those with concurrent HIV and psychiatric comorbidity compared to those with just HIV or psychiatric comorbidity, or neither [64]. A U.S. study of over 7000 adults living with HIV with a median age of 50 years found that metabolic comorbidities were independently associated with depression or anxiety [65]. However, inverse associations between mental health symptoms and BMI among older PWH in some LMIC settings should be noted. In West Africa, with differing social norms and beliefs related to weight and health, PWH with severe depressive symptoms were less likely to be overweight or obese [66]. While the association between unhealthy substance use and dyslipidaemia in our study corroborates others finding an association with alcohol use or smoking [67, 68], positive association between smoking, alcohol or other substance use, and dyslipidaemia among adult PWH has not been consistently identified. Our finding that unhealthy substance use is significantly less likely among those overweight is consistent with many studies in which stimulant and cannabis use are inversely associated with obesity or overweight [69, 70, 71, 72]. While alcohol consumption has been associated with overweight or obesity among PWH, this association has not been consistently found [70, 73]. Although the association between MSDs and increased risk of metabolic comorbidity involves overlapping pathophysiology, behaviours, social determinants of health, systemic inflammation and clinical factors (e.g. medication effects), mood disorders remain predictive of first NCD even after accounting for demographic, behavioural and immunologic co‐founders, and psychiatric medication exposure [63].

While also not found in our cohort, low physical activity levels have been associated with depression, and exercise decreases depression and anxiety symptoms among PWH [74, 75, 76]. The absence of this association in our cohort might relate to unmeasured physical, psychological or social factors that mediate or influence the association, including mobility limitations, sleep impairment, disability, cognition, inflammation, self‐concept, social networks and support [77, 78, 79, 80, 81]. The absence of a significant association between MSDs, type 2 diabetes and hypertension in our cohort is likely explained by differences in the socio‐demographic and HIV clinical profiles of our cohort. Hypertension and diabetes among PWH are associated with older age [82, 83], older PWH experience higher rates of comorbidities and more comorbidities predict greater depression in PWH [84, 85], so the relatively younger age of our cohort, with almost half 40–50 years old, might have attenuated association between MSDs, hypertension or type 2 diabetes. In addition, most of our cohort were on INSTIs, and few on PI‐based regimens, which have been associated with diabetes [86]. Also, we did not assess for some severe mental health disorders such as schizophrenia and bipolar disorder, which might be more strongly associated with NCD comorbidities [87, 88].

Although we did not find an association between liver disease and mental illness symptoms or unhealthy substance use, MSDs have been associated with poor viral hepatitis outcomes, including increased risk of HCV re‐infection, lower likelihood of HCV treatment initiation, worse engagement in HCV‐related care, HCV treatment non‐adherence and increased risk of HCV treatment failure [89, 90, 91, 92, 93, 94]. Alcohol use is also a determinant of liver complications in those with HIV/hepatitis B virus (HBV) co‐infection [95]. A study definition of liver disease covering a variety of underlying liver disease aetiologies potentially shaded associations in our analysis. The associations between MSDs and comorbid NCDs identified in our cohort of older PWH add to calls for sustainable models of care and policies that improve the integration of HIV, MSD and NCD services [63, 96, 97, 98, 99, 100]. Our findings also support expanding approaches and interventions for older PWH in LMICs that address concurrent MSDs and NCDs, including collaborative care, screening, brief interventions and referral, behaviour change interventions, and health promotion and literacy interventions [101, 102]. With most research from Western and high‐income country PWH cohorts, additional research on the associations between MSDs and comorbidities among older PWH, their impacts and care integration approaches in LMIC settings are also warranted. Improved integration approaches are particularly important given the suboptimal levels of integration of HIV, MSD, and co‐morbidity screening and treatment services often found in resource‐constrained settings [103, 104, 105, 106].

Our findings should be interpreted in the context of some limitations. First, our study sample is of PWH aged ≥40 on ART in care mostly at relatively well‐resourced sites, raising the potential for sampling bias, lower rates of clinical outcomes, better HIV‐related outcomes and limiting the generalizability of our findings to the broader population of older PWH in a particular country. Second, the analysis methodology does not support a causal assessment of MSDs and comorbidities, nor the assessment of effect heterogeneity by geographical region or sex. Third, because our analysis used a combined mental illness symptom and unhealthy substance use outcome, we were not able to differentiate between different types of mental illness or substances. Fourth, the performance of translated MSD screening tools across all study settings was not formally assessed through psychometric validation prior to study screenings. Last, social desirability and recall bias might also have resulted in underreporting of mental illness symptoms and substance use. Despite these limitations, this analysis provides informative data on MSDs and associated comorbidities, and is one of very few from a global cohort of older PWH in LMICs.

## 5 CONCLUSIONS

The high prevalence of comorbidities among those with mental illness symptoms or unhealthy substance use in our global cohort of PWH ≥40 years old, and the association of mental illness symptoms or unhealthy substance use with older age, underweight BMI or dyslipidaemia highlight the need for improved MSD and comorbidity screening, diagnosis and treatment services as PWH age, and improved service integration in HIV clinical settings in LMICs. Further research is needed on the association between MSDs and comorbidities, the impacts of MSDs on comorbidity outcomes and care integration approaches among older PWH in LMIC settings. Enhanced policy support to service integration is also required.

## COMPETING INTERESTS

The authors declare that they have no competing interests.

### AUTHORS’ CONTRIBUTIONS

JLR developed the research concept with support from DR, TC, AP, KL and AS. DR analysed the data. JLR interpreted the data and drafted the manuscript, with input from DR. TC, BCR, GM, FM, AM, IM, HP, AP, SG, CC, EM, FM, DTHN, AL‐I, KL, HB, MT, MKP, RM, AS, GW, AJ and AS reviewed manuscript drafts. All authors have read and approved the final manuscript.

## FUNDING

The International Epidemiology Databases to Evaluate AIDS (IeDEA) is supported by the U.S. National Institutes of Health's National Institute of Allergy and Infectious Diseases, the *Eunice Kennedy Shriver* National Institute of Child Health and Human Development, the National Cancer Institute, the National Institute of Mental Health, the National Institute on Drug Abuse, the National Heart, Lung, and Blood Institute, the National Institute on Alcohol Abuse and Alcoholism, the National Institute of Diabetes and Digestive and Kidney Diseases, and the Fogarty International Center: Asia‐Pacific, U01AI069907; CCASAnet, U01AI069923; Central Africa, U01AI096299; East Africa, U01AI069911; Southern Africa, U01AI069924; West Africa, U01AI069919. Informatics resources are supported by the Harmonist project, R24AI24872.

## DISCLAIMER

This work is solely the responsibility of the authors and does not necessarily represent the official views of any of the institutions mentioned above.

## Data Availability

Complete data for this study cannot be posted in a supplemental file or a public repository because of legal and ethical restrictions. The Principles of Collaboration under which this multi‐national consortium was founded and the regulatory requirements of the different countries’ IRBs require the submission and approval of a project concept sheet. The data held by the IeDEA consortium are available to other investigators, but must be based on a concept sheet describing the planned analysis, and approved by the regional Steering Groups and, if analyses involve several regions, by the IeDEA Executive Committee (https://www.iedea.org/working‐groups/executive‐committee/). Additional information is available online at https://www.iedea.org/resources/.
